# Impact of Salinity on Rice Production in Vulnerable Southern Coastal Regions of Bangladesh: A Case Study Through a Cobb–Douglas Stochastic Production Function Model

**DOI:** 10.1002/pei3.70233

**Published:** 2026-10-28

**Authors:** Shahin Imran, Newton Chandra Paul, Md. Asif Mahamud, Jotirmoy Chakrobortty, Md. Najmol Hoque, Md Shihab Uddine Khan, Md. Jahurul Islam, Md. Rasheduzzaman, Akbar Hossain

**Affiliations:** ^1^ Department of Agronomy Khulna Agricultural University Khulna Bangladesh; ^2^ Department of Agricultural Chemistry Khulna Agricultural University Khulna Bangladesh; ^3^ Department of Soil Science Khulna Agricultural University Khulna Bangladesh; ^4^ Department of Biochemistry and Molecular Biology Khulna Agricultural University Khulna Bangladesh; ^5^ Plant Physiology Division Bangladesh Agricultural Research Institute Gazipur Bangladesh; ^6^ Department of Agricultural Economics Khulna Agricultural University Khulna Bangladesh; ^7^ On‐Farm Research Division Bangladesh Agricultural Research Institute Sylhet Bangladesh; ^8^ Agronomy Division Bangladesh Wheat and Maize Research Institute Bangladesh

**Keywords:** Bangladesh, coastal areas, migrating farmers, rice production, salinity stress

## Abstract

Salinity in coastal areas has a significant effect on the technical efficiency (maximum potential yield utilizing available inputs) of rice production and subsequently reduces food security in developing countries. We examined the salinity status and its impact on overall rice production across seasonal cycles in the southern coastal region of Bangladesh, utilizing data from 464 farmers from vulnerable coastal areas. The estimation from the Cobb–Douglas stochastic production function revealed that salinity has a statistically negative relationship with rice yield. Our study revealed that technical efficiency increased when farmers adopted salt‐tolerant rice varieties and special techniques to manage salinity problems. Consequently, the profit per hectare was greater in the low‐salinity zone than in the moderate‐ and high‐salinity zones. We also find from binary logistic regression that with a greater possibility of yield loss, rice quality destruction, and salt land, farmers were more likely to migrate from cultivated rice to other crops because of salinity problems. In contrast, higher income, more consultations with extension officers and other farmers, and training knowledge, the likelihood of migrating from rice cultivation is lower. To mitigate salinity problems and ensure a sustainable future, salt‐tolerant hybrid rice varieties need to be developed by research institutes, and frequent consultation and training need to be arranged by extension departments in Bangladesh.

## 1 Introduction

Globally, salinity is a significant challenge to coastal agricultural production, especially in areas where sustainable agricultural production is the primary need for economic stability and food security (Rashid et al. 2023; Oelviani et al. 2024). The devastating effects of salinity on staple crops such as rice are well known in South and Northeast Asia. Rice production is hampered by salinity (20%–30%) in Bangladesh (Islam, Bruyn, et al. 2021). Therefore, considering climate change, soil salinity is a serious issue (Chele et al. 2021). It accounts for approximately 20% of the world's cultivated area (Shahid et al. 2018) and is expected to increase to 50% of all arable land by 2050 because of climate change in the future (Wang et al. 2003). Considering this fact, Southeast Asia should mitigate salinity issues by using advanced methods and materials to ensure robust agricultural productivity, food security, and economic stability.

Bangladesh is a deltaic Southeast Asian country bordered in the south by the Bay of Bengal (Hoque et al. 2021). Approximately 28% of district areas (18 districts out of 64) in the southwest region are recognized as being affected by salinity (SRDI 2010a). In terms of area, it affects approximately 20% (0.22 million hectares) of the country's territory, which encompasses 30% of the cultivated area and is located in the southwestern part of Bangladesh (Hasan et al. 2019). Among approximately 3.072 million hectares of coastal land in Bangladesh, 1.689 million hectares are cultivated areas, of which 1.056 million hectares (approximately 63%) are affected by various degrees of salinity (SRDI 2010a). In addition, approximately 27% of new land has been affected by varying degrees of salinity during the previous four decades (SRDI 2010a; Hasan et al. 2019). Moreover, salinity in coastal areas has increased because of upstream freshwater flow, tidal flooding, and storm surges, negatively impacting the country's overall rice production (Guimond and Michael 2021; Rahman and Uddin 2021).

Rice production accounts for approximately 78% of the total net cultivated area in Bangladesh (Mamun et al. 2021). It has recently become the third most common form worldwide in terms of rice production. The climate of Bangladesh is ideal for rice cultivation, which is consumed by approximately 160 million Bangladeshis (Singha et al. 2021). In Bangladesh, food security is equivalent to rice security (Mamun et al. 2021). However, rice production is primarily harmed by biotic and abiotic stresses, which result in a complex interplay between stress factors and a variety of physiological, molecular, and biochemical processes that affect plant growth and development (Tahjib‐Ul‐Arif et al. 2021). In addition, climate change affects Bangladesh, and salt stress is among the key stressors that affect rice production through the modification of metabolism and gene expression, the reduction of water potential and ionic imbalance, the increase in toxicity, and the subsequent disruption of plant growth and development (Jodder et al. 2016; Chele et al. 2021). Rice production efficiency is highly sensitive to salinity stress, with a tolerable range of 3 dSm^−1^ for most field varieties (USDA 2013). Therefore, salinity could impact the technical efficiency, which is the ability to produce the maximum possible yield within a given set of inputs of rice production across all seasons in the southern coastal areas of Bangladesh. In addition, this adverse effect could force farmers to migrate toward crops other than rice or even other professions.

There is a wealth of previous literature on the effects of salinity on agriculture in Bangladesh. Most of them are categorized into the following categories: the extent and causes of salinity in coastal areas (Khanam et al. 2020; Miah et al. 2020; Alam and Hasan 2023), the effects on crop production (Miah et al. 2020; Islam et al. 2023), shifts in cropping patterns (Hajong et al. 2021; Islam et al. 2023; Rashid et al. 2023; Badhan et al. 2024), challenges in addressing salinity (Hoque et al. 2021; Islam, Alam, et al. 2021; Rahman and Uddin 2021; Bhuyan et al. 2024), and adaptive strategies and mitigation practices (Kumar et al. 2018; Hossain and Li 2024; Haque et al. 2025). However, studies on the technical efficiency of rice production under saline conditions in Bangladesh are limited. A study has compared the technical efficiency of rice production in saline and non‐saline areas of Bangladesh (Sarker et al. 2022). In contrast, salinity intrusion significantly reduces rice yields, leading farmers to seek alternative crops (Nguyen et al. 2024; Kabir et al. 2024). In Bangladesh, smaller farms are shifting toward vegetables, whereas larger farms are adopting high‐value crops such as watermelon because of the detrimental effects of salinity on rice (Kabir et al. 2024). Farmers are increasingly adopting salt‐tolerant crop varieties and conservation practices, such as zero tillage and crop rotations, which have been shown to improve soil health and crop yields (Sikder and Khan 2024). In Vietnam, switching to crops such as vegetables and shrimp has been linked to higher revenues and improved food security (Nguyen et al. 2024). When adaptation strategies are insufficient, farmers may resort to migrating inland to escape the impacts of salinity and flooding, as evidenced in Mozambique (Bruijn and Liaw 2023). On the bottom line, a literature review confirms that studies on the technical efficiency of rice production under saline conditions and the effects of salinity intrusion on the migration of rice farmers to other crops are limited.

Despite the extensive literature on coastal salinity, crop shifts, challenges, and adaptation strategies, significant research gaps remain. First, very few empirical studies have evaluated how different levels of salinity alter the technical efficiency of rice production using stochastic frontier modeling. Second, while the migration of farmers toward other crops has been identified qualitatively, an econometric evaluation of how salinity drives farmers' decisions to shift from rice to alternative high‐value crops remains underexplored in the southern coastal region of Bangladesh. Therefore, the novelty lies in combining these two issues in an integrated assessment of salinity on farm level technical efficiency and crop choice behavior. Finally, it is hypothesized that increased salinity severity significantly affects the technical efficiency of rice production and drives farmers' decisions to shift away from rice toward alternative, salt‐tolerant crops. Under these circumstances, this study attempts to quantify the effects of salinity on the technical efficiency of rice production and on the migration of rice farmers to other crops in the southern coastal area of Bangladesh.

## 2 Materials and Methods

### 2.1 Study Area and Sampling Design

In this study, the authors chose to work in the southern coastal areas of Bangladesh. Eighteen districts in the southwestern region of Bangladesh (Satkhira, Khulna, Bagerhat, Pirojpur, Jhalakathi, Barisal, Bhola, Patuakhali, Borguna, Narail, Jessore, Madaripur, Gopalganj, Laxmipur, Noakhali, Feni, Chittagong, and Cox's Bazar) have been identified as salinity‐prone areas (SRDI 2010b). A multistage sampling technique was utilized to select representative farming households from the southern coastal zone of Bangladesh. In the first stage, three coastal districts—Khulna, Bagerhat, and Satkhira—were selected according to the high severity of salinity‐affected rice cultivable land (approximately 33%–41%) (SRDI 2010b; Hasan et al. 2019). In the second stage, a total of 12 upazilas exhibiting different levels of salinity were selected based on expert consultations: five upazilas from Khulna (Dumuria, Dacope, Batiaghata, Rupsa, and Paikgachha), three from Bagerhat (Fakirhat, Mongla, and Rampal), and four from Satkhira (Assasuni, Kaliganj, Shyamnagar, and Tala) (Figure 1).

**FIGURE 1:**
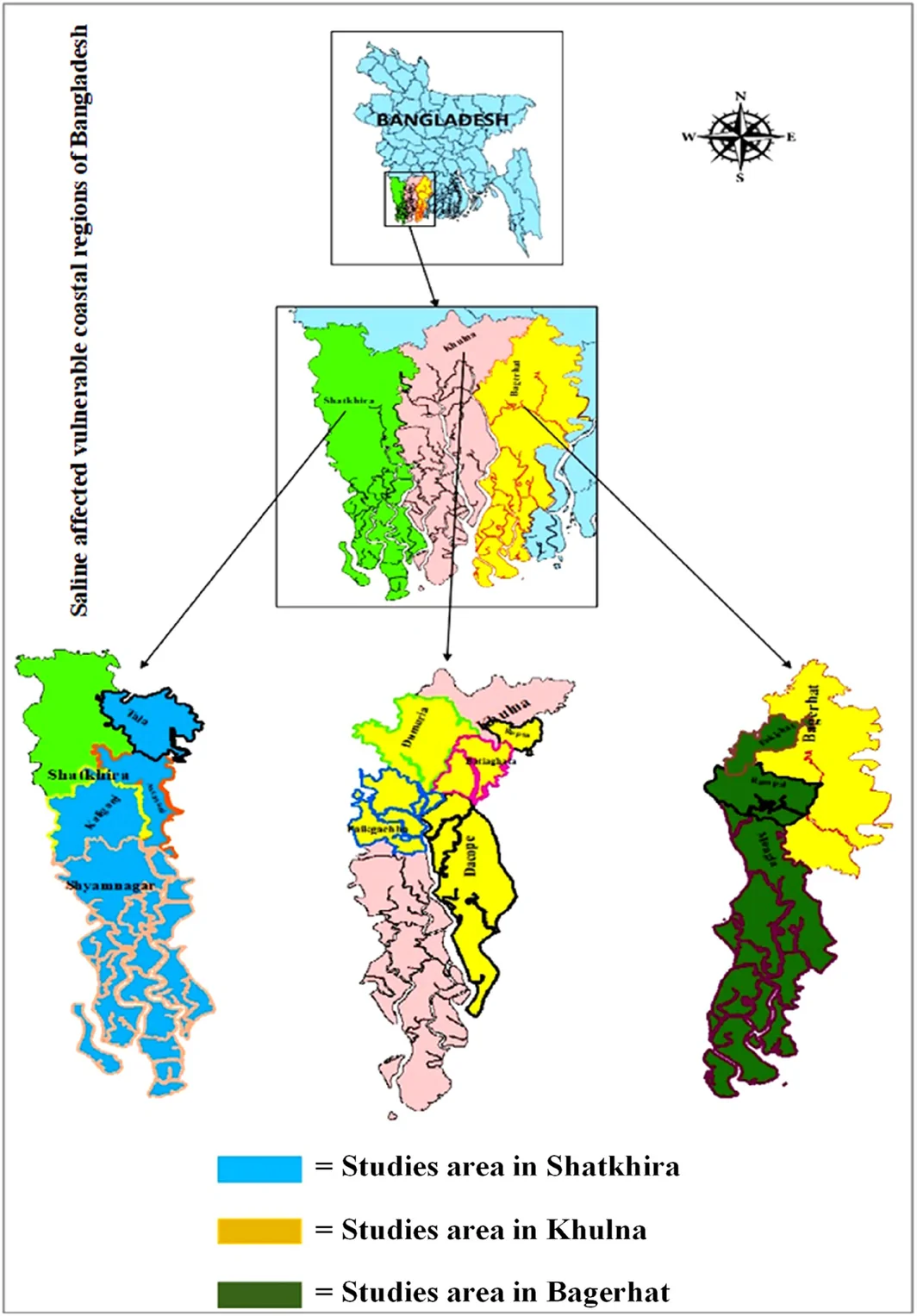
Map covering the southern coastal region of Bangladesh.

In the third and final stage, the study identified the sample and recorded the data through a face‐to‐face structured questionnaire. Farmers who grow rice as their primary crop were included in this study's population. However, the impact of salinity on rice production does not necessarily focus on any specific cropping seasons. The survey was conducted to measure the impact of salinity on rice production efficiency irrespective of the cropping season. From the study area, a sample of 464 rice‐producing farmers was randomly selected using a draft list provided by the Upazila Agricultural Extension Offices.

In accordance with the study's objectives, a structured interview schedule was created. It was then pretested among a few farmers who were not included in the sample. Primary data from rice‐producing farmers were collected through face‐to‐face interviews on socioeconomic characteristics, rice production practices, various inputs used, the effects of salinity on rice cultivation, and their knowledge of salinity management.

### 2.2 Technical Efficiency Analysis of Rice Production in Coastal Areas

Several studies related to the salinity impact and adaptation context have considered the Cobb–Douglas production function model (Basu and Roy 2018; Islam 2018). The model has been applied to evaluate the effects of salinity on rice yield (Ahmed and Haider 2014). The stochastic frontier production function, introduced autonomously by Aigner et al. (1977) and Meeusen and van Den Broeck (1977), substantially affects econometric modeling and the assessment of farm technical efficiency. A stochastic frontier model was employed to assess the yield and efficiency level of the rice producers. We adopted the following production function model to assess the impact of salinity and the use of other inputs on rice yield in the southern coastal area of Bangladesh.

(1) $$\ln\left(Y\right)=\alpha_{0}+\alpha_{1}\ln\left(L\right)+\alpha_{2}\ln\left(S\right)+\alpha_{3}\ln\left(F\right)+\alpha_{4}\ln\left(H\right)+\alpha_{5}\left(\text{Urea}\right)+\alpha_{6}\;\left(\mathrm{TSP}\right)+\alpha_{7}\left(\mathrm{MOP}\right)+\alpha_{8}D_{1}+\alpha_{9}D_{2}+\alpha_{10}D_{3}+\varepsilon_{i}$$

Here, *Y* = rice yield (kg/decimal; where 1 ha = 247.105 decimals), *L* = cost of labor used, *S* = seed used, *F* = fertilizer cost, *H* = harvesting cost, urea, TSP and MOP refer to the usage of fertilizer, and *D*_1_, *D*_2_, and *D*_3_ represent the dummy variables for the level of salinity, where *D*_1_ is for the low‐salinity region, *D*_2_ is for the moderately saline region, and *D*_3_ is for the high‐salinity region. The technical inefficiency model is defined as follows:

(2) $$U_{i}=\delta_{0}+\delta_{1}X_{1}+\delta_{2}X_{2}+\delta_{3}X_{3}+\delta_{4}X_{4}+\delta_{5}X_{5}+\delta_{6}X_{6}+W_{i}$$

Here, *U*_*i*_ is the inefficiency, *δ* is the parameter, *X*_1_ is farming experience, *X*_2_ is the high price of labor, *X*_3_ is the use of salt‐tolerant rice varieties, *X*_4_ is training knowledge, *X*_5_ is consultation with AEO, and *X*_6_ refers to the special techniques used to manage salinity.

Following the technical efficiency of rice production, the study also assessed farm performance. The performance level of the farmers was assessed to identify the variations in the level of salinity and production of rice yield (Ahmed and Haider 2014). A linear functional form is calculated below to reflect the relationship of the farmers' performance with varied amounts of saline‐affected land and land size, migrating from rice cultivation land to other crops, and total income as explanatory variables to evaluate the performance of the farmers involved in rice cultivation in the research areas.

(3) $$P=\beta_{0}+\beta_{1}\mathrm{CL}+\beta_{2}\mathrm{ML}+\beta_{3}\mathrm{TI}+\beta_{4}D_{1}+\beta_{5}D_{2}+U$$

Here, *P* is the farmers' performance, which is measured as the ‘profit‐to sales’ ratio, and Profit is measured as the net return, which is the sales minus the cost of production on the same land. The sales value is calculated by multiplying the yield of rice (kg/decimal) and the price of the rice (BDT/kg). Here, CL is the total cropland or size of the land expressed in hectares, ML is the amount of land migrating to other crops from rice cultivation land because of salinity problems, TI is the total income reflected as the performance for the farm, and *D*_1_ and *D*_2_ are salinity‐related dummy variables (where *D*_1_ for moderately saline regions and *D*_2_ for highly saline regions) as the independent variables.

### 2.3 Binary Logistic Regression for the Migration of Rice to Other Crops

To assess the determinants influencing the migration of cultivated rice to other crops because of salinity problems, logit or probit models can be applied to elucidate the categorical or continuous variables (Wooldridge 2015). Several studies have applied binary models to identify the factors or determinants affecting the migration of cultivated rice to other crops because of salinity issues (Anisuzzaman et al. 2016; Chen and Mueller 2018). In this binary model, our interest lies primarily in the response probability (Wooldridge 2015), according to the following equations:

(4) $$P\left(\;y=1|x\right)=P\left(\;y=1|x_{1},x_{2},\ldots ,x_{\text{k}}\right)$$

Here, *x* refers to the complete set of independent variables, while *y* is an indicator of migration from rice to other crops. More specifically, we look at the concept of the binary logistic model, which considers the factors that force the migration of cultivated rice to other crops.

(5) $$\begin{array}{c} P\left(Y=1|X\right)=b_{0}+b_{1}X_{1}+b_{2}X_{2}+b_{3}X_{3}\\ \;+b_{4}X_{4}+b_{5}X_{5}+b_{6}X_{6}+b_{7}X_{7}+b_{8}X_{8}+b_{9}X_{9} \end{array}$$

Here, *Y* represents the probability of migration from rice to other crops because of salinity (1 if migrated, 0 otherwise), *X*_1_ = age of the farmer, *X*_2_ = total crop land, *X*_3_ = plant death, *X*_4_ = yield loss, *X*_5_ = destruction of quality of rice, *X*_6_ = farming experience, X_7_ = salt land, *X*_8_ = total income, X_9_ = consulting with AEOs, *X*_10_ = consulting with other farmers, *X*_11_ = training knowledge, *X*_12_ = level of experience, and *X*_13_ = any special technique to manage salinity.

### 2.4 Rank Order of Barriers Faced in Terms of Rice Cultivation in Coastal Areas

Finally, the rank order of the barriers that farmers faced in terms of cultivating rice was calculated considering the salinity problem in coastal areas of Bangladesh. The frequency and percentages of responses were recorded in this section. Following these barriers, the study also suggested some recommendations to overcome those problems.

## 3 Results and Discussion

### 3.1 Descriptive Statistics of the Social, Economic, and Demographic Variables

The current salinity status and its impact on rice production is an upsetting incident in the southern coastal area of Bangladesh. High salinity persists in these regions of Bangladesh, namely, Khulna, Satkhira, Patuakhali, Cox's Bazar, Barguna, Bhola, Noakhali, Lakshmipur, and Chattagram. The descriptive statistics of the variables applied in this study are listed in Table 1. Some differences in the particular variables (age, farming experience, cost of fertilizer, use of fertilizer, level of salinity, use of salt‐tolerant rice variety, knowledge of training, consultation with extension officers, total crop land, yield loss, destruction of rice quality, salt land, total income, and special techniques to manage salinity) were not significant, whereas the differences in the other variables were not significant. The statistics of the social, economic, and demographic characteristics are presented in Table 1.

**TABLE 1:** Descriptive statistics of the variables.

Variables (unit)	Mean	Standard deviation	Minimum	Maximum
Age (years)	43.993	12.200	20	90
Education (years)	5.491	5.464	0	18
Family size (number)	5.181	1.956	2	28
Farming experience (years)	22.637	11.755	2	60
Rice cultivation (years)	21.961	11.691	2	60
Total cropland (decimal)	188.960	177.722	16.50	1485
Saline‐affected cropland (decimal)	133.310	173.101	0	1485
Level of salinity (level)	1.385	0.854	0	3
Migrating from rice cultivation (yes/no)	0.295	0.457	0	1
Migrating land (%)	6.158	15.976	0	80
Labor cost (BDT/decimal)	38.250	23.025	0	96.970
Seed cost (BDT/decimal)	24.688	19.721	0	121.212
Fertilizer cost (BDT/decimal)	37.869	16.886	0	81.818
Harvesting cost (BDT/decimal)	42.913	20.1015	0	90.909
Urea (Kilogram/decimal)	0.987	0.735	0	3.788
TSP (Kilogram/decimal)	0.325	0.265	0	1.512
MoP (Kilogram/decimal)	0.245	0.178	0	0.909
Herbicides (yes/no)	0.297	0.458	0	1
Total income (BDT/decimal)	491.330	91.547	30.91	824.24
Yield (Kilogram/decimal)	18.530	3.047	12.12	36.36
Plant death (yes/no)	0.203	0.402	0	1
Yield loss (yes/no)	0.797	0.402	0	1
Destroy quality of rice (yes/no)	0.440	0.497	0	1
Salty land (yes/no)	0.619	0.486	0	1
High price of labor (yes/no)	0.791	0.438	0	1
Lack of capital (yes/no)	0.625	0.485	0	1
Lack of extension services (yes/no)	0.483	0.500	0	1
Lack of seeds (yes/no)	0.140	0.347	0	1
High price of seeds (yes/no)	0.627	0.497	0	1
High price of pesticide (yes/no)	0.558	0.497	0	1
High price of labor (yes/no)	0.791	0.438	0	1
Shortage of fertilizer (yes/no)	0.151	0.358	0	1
Knows from training (yes/no)	0.112	0.316	0	1
Knows from experience (yes/no)	0.694	0.461	0	1
Consulting with AEO (yes/no)	0.481	0.500	0	1
Consulting with other farmers (yes/no)	0.847	0.360	0	1
Any special technique to manage salinity (yes/no)	0.485	0.500	0	1

### 3.2 Technical Efficiency of Rice Production in Coastal Areas

To calculate the efficiency level of the farmers, several variables, such as labor, seed, fertilizer, harvesting costs, and use of fertilizer (i.e., urea, TSP, MOP, and herbicide), and the level of salinity were used. Table 2 shows the Cobb–Douglas stochastic production frontier parameters computed using the maximum likelihood estimation approach. The findings indicate that the cost of fertilizer and fertilizer usage (i.e., TSP and MOP) have a considerable and significant effect on rice yield. Therefore, the higher the cost and input used (i.e., fertilizer), the higher the rice yield. Similar analyses have been performed by Sultana et al. (2022), who reported that inputs such as labor, seed, urea, and insecticide significantly affect the level of production of Aman rice. It is also evident from the results of this study that when the level of salinity is low, the yield of rice increases significantly (Nicolas et al. 2023). Therefore, high‐salinity areas are more vulnerable to rice production than low‐ and moderate‐salinity areas are in Bangladesh. The impact of salinity on the technical efficiency of rice cultivation is presented.

**TABLE 2:** Estimation of the Cobb–Douglas stochastic production function and the effect of technical inefficiency on rice yield.

Dependent variable: Ln (Y): yield	Coefficient	Standard error
Ln (L): labor	0.001	0.005
Ln (S): seed	−0.007	0.008
Ln (F): fertilizer	0.048***	0.016
Ln (H): harvesting	−0.002	0.006
Urea	0.016	0.013
TSP	0.071**	0.031
MoP	0.177***	0.048
Herbicides	−0.004	0.017
*Level of salinity*		
Moderate saline	0.043*	0.022
High saline	−0.008	0.030
Constant	2.663***	0.061
*Inefficiency model*		
Farming experience	0.002**	0.001
High price of labor	−0.021	0.017
Use of salt‐tolerant rice variety	−0.059***	0.015
Knows from training	0.094***	0.023
Consulting with AEO	0.051***	0.017
Any special technique to manage salinity	−0.081***	0.016
Constant	3.045***	0.029
Variance parameter		
Sigma square	0.037***	0.007
Mean efficiency	0.895	

* *p* < 0.1 level of significance.

** *p* < 0.05 level of significance.

*** *p* < 0.01 level of significance.

Table 2 also reflects the findings of the inefficiency, which expresses the role of social, economic, and demographic variables in the technical efficiency of rice production. This function of inefficiency is explained by six variables, such as farming experience, high labor price, use of salt‐tolerant rice varieties, knowledge from training, consultation with an extension officer (AEO), and any special techniques to manage the salinity. The findings show that using a salt‐tolerant rice variety and applying special techniques to manage salinity have a statistically significant effect on technical inefficiency. This implies that if farmers adopt salt‐tolerant rice varieties and special techniques to manage salinity problems, their efficiency will increase because of their informed knowledge of the salt‐tolerant rice variety and experience with the use of special techniques to address salinity issues (Rahman et al. 2024; Sikder and Khan 2024). However, despite having knowledge of training and consultation with extension officers, their technical efficiency would not increase because of the salinity problem in the study area. The entire mean efficiency was 89.52%, implying that by optimizing inputs, nearly 11% of the yield gap might Sultana et al. (2022) narrow. In their study, reported opposite findings, indicating that farmers' access to credit and training is statistically significantly related to technical inefficiency, implying that when farmers receive training and credit for rice production, their efficiency increases as a result of their learning about new technology and knowledge from training that can be utilized in the field. This is apposite because their study did not consider the issue of salinity when the efficiency level was measured.

### 3.3 Performance of Rice Cultivation in Terms of Salinity

Salinity intrusion exerts a detrimental effect on agricultural productivity. In this study, the profit–sales ratio is employed to assess the performance of the farm. Improved farm productivity is closely linked to increased net return or profit per decimal. Because rice yields are lower in areas with high salinity and higher in low‐salinity zones, profitability varies accordingly. Consequently, as detailed in Table 3, profit per decimal in low‐salinity areas is significantly greater than that in moderate‐ and high‐salinity regions.

**TABLE 3:** Indicators of farm performance.

Indicators	Low saline area		Moderate saline area		High saline area	
	Mean	CV (%)	Mean	CV (%)	Mean	CV (%)
Profit (BDT/decimal)	237.410	51.510	203.613	56.990	190.380	44.950
Sales (BDT/decimal)	497.150	18.700	491.698	19.920	462.260	17.870
Performance	0.456	40.780	0.346	184.970	0.396	36.610
Land size/total crop land (ha)	0.706	71.670	0.878	102.270	0.938	109.70

*Note:* Performance = Profit–Sales ratio, and 247.105 decimal = 1 ha.

Land size and salinity level influence the performance of a farm. The calculated *R* ^2^ indicates that 24% of the total variation in performance is explained by the variation in the level of salinity, total income, and migration from cultivated rice to other crops due to salinity (Table 4).

**TABLE 4:** Determinants of farm performance.

Farm performance (dependent variable)	Coefficient	Standard error
Crop land or land size (hectare)	−0.010	0.023
Migrating from rice cultivation due to salinity	0.115***	0.042
Total income	0.002***	0.001
Level of salinity		
Low saline area	−0.059	0.049
Moderate saline region	−0.179***	0.053
High saline region	−0.121*	0.073
Constant	−0.532***	0.099
*R*‐squared	0.243	

* *p* < 0.1 level of significance.

*** *p* < 0.01 level of significance.

The computed regression coefficients of the salinity levels clearly reveal a negative relationship between the salinity level and farm performance, and this association was statistically significant (Table 4). The coefficients of moderate (*D*_1_) and high (*D*_2_) salinity (*β*_4_ = −0.17 and *β*_5_ = −0.12), in particular, demonstrate a statistically significant inverse relationship between agricultural productivity and salinity level. Migrating from rice cultivation to other crops because of the farmer's salinity and higher total income has a significant positive effect on the productivity of agriculture. Finally, the study reveals that compared to low‐salinity areas, the profit‐sales ratios in moderate‐ and high‐salinity regions are 17% and 12% lower, respectively.

Similar studies were conducted by Ahmed and Haider (2014), who reported that in highly saline regions, the amount of rice produced per hectare is lower, and vice versa. Compared with moderate‐ and high‐salinity regions, profit per hectare is greater in low‐salinity regions (Nicolas et al. 2023). An estimated regression coefficient is inversely related to the level of salinity (Russ et al. 2020; Thach et al. 2023).

### 3.4 Migration of Rice Farmers to Other Types of Crop Cultivation due to Salinity

The data in Table 5 reflect the factors driving the migration from rice cultivation to alternative crops due to high salinity in the fields. The marginal effect from the binary logistic regression is explained by the number of variables, such as age, total cropland, plant death, loss of yield, salt land, total income, consulting with the other farmers and the extension officers, and knowledge from the training.

**TABLE 5:** Marginal effects of the factors influencing the migration of cultivated rice to other crops due to salinity.

Dependent variable	Coefficient	Standard error
Age	0.005**	0.002
Total crop land	0.001***	0.001
Plant death	0.033	0.052
Yield loss	0.217***	0.059
Destroy the quality of rice	0.115***	0.040
Farming experience	0.001	0.003
Salty land	0.267***	0.040
Total income	−0.001***	0.001
Consulting with AEO	−0.189***	0.041
Consulting with other farmers	−0.178***	0.055
Knows from training	−0.152**	0.077
Knows from experience	−0.036	0.040
Any special technique to manage Salinity	−0.046	0.047

** *p* < 0.05 level of significance.

*** *p* < 0.01 level of significance.

Our model reveals that age, total cropland, yield loss, rice quality destruction, and salt land have positive and significant effects on migration from cultivated rice to other crops. This implies that with increasing age, the possibility of yield loss, rice quality destruction, and salt land is more likely to migrate from cultivated rice to other crops because of salinity problems. In contrast, total income, consultation with AEOs and other farmers, and knowledge from training have negative and significant effects on the migration from cultivated rice to other crops because of salinity. These findings suggest that higher income, frequent extension consultations, and better training reduce the likelihood migrate from rice to other types of crop cultivation.

Our findings are corroborated by the study of Chen and Mueller (2018), where they reported that farming households move toward other types of farming, such as aquaculture, as the issue of salinity hinders the production of rice and crop farming. Furthermore, at least in terms of agricultural production, this replacement appears to be successful in mitigating income risk due to flooding and soil salinity (Chen and Mueller 2018). Anisuzzaman et al. (2016) reported that several factors, such as land holding, age, occupation, and intrusion of saline water, were significantly related to migration from cultivated rice to cultured shrimp in the southwestern region of Bangladesh. The possibility of yield loss for farmers cultivating rice in saline areas is a significant concern, as salinity adversely affects crop productivity (Dam et al. 2019). Kabir et al. (2024) reported that the risk of yield loss influences farmers' decisions to avoid cultivating rice in saline areas and opt for alternative crops such as vegetables. Furthermore, the risk of quality deterioration in rice due to salinity, particularly during the sensitive regreening to panicle initiation stage, may compel farmers to choose alternative crops in saline areas to ensure better yield and quality outcomes (Ismail et al. 2019; Li et al. 2023). Farmers tend to opt for crops other than rice if high salt is present in the cropland because high salt concentrations can disrupt nutrient uptake, resulting in deficiencies that further impair crop yields (Teshale 2023).

In contrast, farmers with higher incomes tend not to change their rice cultivation, as it can harm them less economically. They could adopt a salinity‐resistant rice variety and management practices against salinity, as they have enough economic stability. Because of the higher return possibility, they even use rice cultivation in saline areas (Rahman et al. 2013). Farmers continue to cultivate rice under saline conditions and often rely on consultations with other farmers and extension agents to adapt their practices. This ongoing cultivation is influenced by several factors, including the development of salt‐tolerant rice varieties, improved irrigation techniques, and collaborative efforts among stakeholders (Mills et al. 2022). Accordingly, despite the challenges posed by salinity, many farmers have gained knowledge through training and have implemented various practices to mitigate its effects on rice cultivation (Sikder and Khan 2024).

### 3.5 Barriers to Rice Cultivation and Ways of Managing Salinity in Coastal Areas

There are several major constraints to rice cultivation identified by the farmers in the southern coastal region of Bangladesh (Table 6). Among these constraints, farmers rank high labor cost as the main obstacle to rice cultivation. Other major constraints include a lack of capital, a high price of seeds, salt land, a high price of inputs, a lack of extension services, a dearth of fertilizer usage, and a lack of seeds. Owing to the high wage rate of labour, the price of inputs, and high salinity issues, farmers are discouraged from rice cultivation, which is corroborated by several studies (Islam 2018; Islam et al. 2017; Suvi et al. 2020).

**TABLE 6:** Constraints to rice cultivation in coastal areas.

Constraints to rice cultivation	Frequency		Percentage of farmers faced the constraints	Ranking of the problem
	Yes	No		
High price of labor	364	100	78.45	1
Lack of capital	290	174	62.50	2
High price of seeds	288	176	62.07	3
Salty land	287	177	61.85	4
High price of pesticide/herbicide	259	205	55.82	5
Lack of extension services	224	240	48.28	6
Shortage of fertilizer	70	394	15.09	7
Lack of seeds	65	399	14.01	8

Salinity influx has already harmed rice output in the Satkhira area of Bangladesh (Rabbani et al. 2013). Furthermore, logistical difficulties, such as a lack of irrigation infrastructure during the dry season and geography, which affects flood depth and soil salinity in coastal areas, were important barriers to the adoption of contemporary rice varieties (Hossain et al. 2006). Disease, insect pests, recurrent drought, nonavailability and high‐cost fertilizers, a lack of improved cultivars, and poor soil fertility were key restrictions affecting rice output and productivity at all the examined locations (Suvi et al. 2020). Moreover, poor infrastructure, high input costs, credit problems, low rice prices, inadequate inputs, and a lack of training were identified as the most significant socioeconomic obstacles assessed by a substantial number of farmers (Thanh and Singh 2006). Shelley et al. (2016) reported several factors that affect rice cultivation, such as drought, flood, salinity, extreme temperature, soil fertility, various pests and insects, and multiple stresses (i.e., early monsoon and extreme flooding).

There are several techniques followed by farmers in managing the salinity of crop land in the coastal areas of Bangladesh. Among the techniques adopted, our study revealed that approximately 85% of the farmers reported that they consulted with other farmers who had more farming experience, 70% of the farmers utilized their own farming experience in managing the salinity of the cropland, 49% of the farmers followed several special techniques to manage the salinity, and 48% of the farmers consulted with local extension officers regarding the salinity management (Figure 2).

**FIGURE 2:**
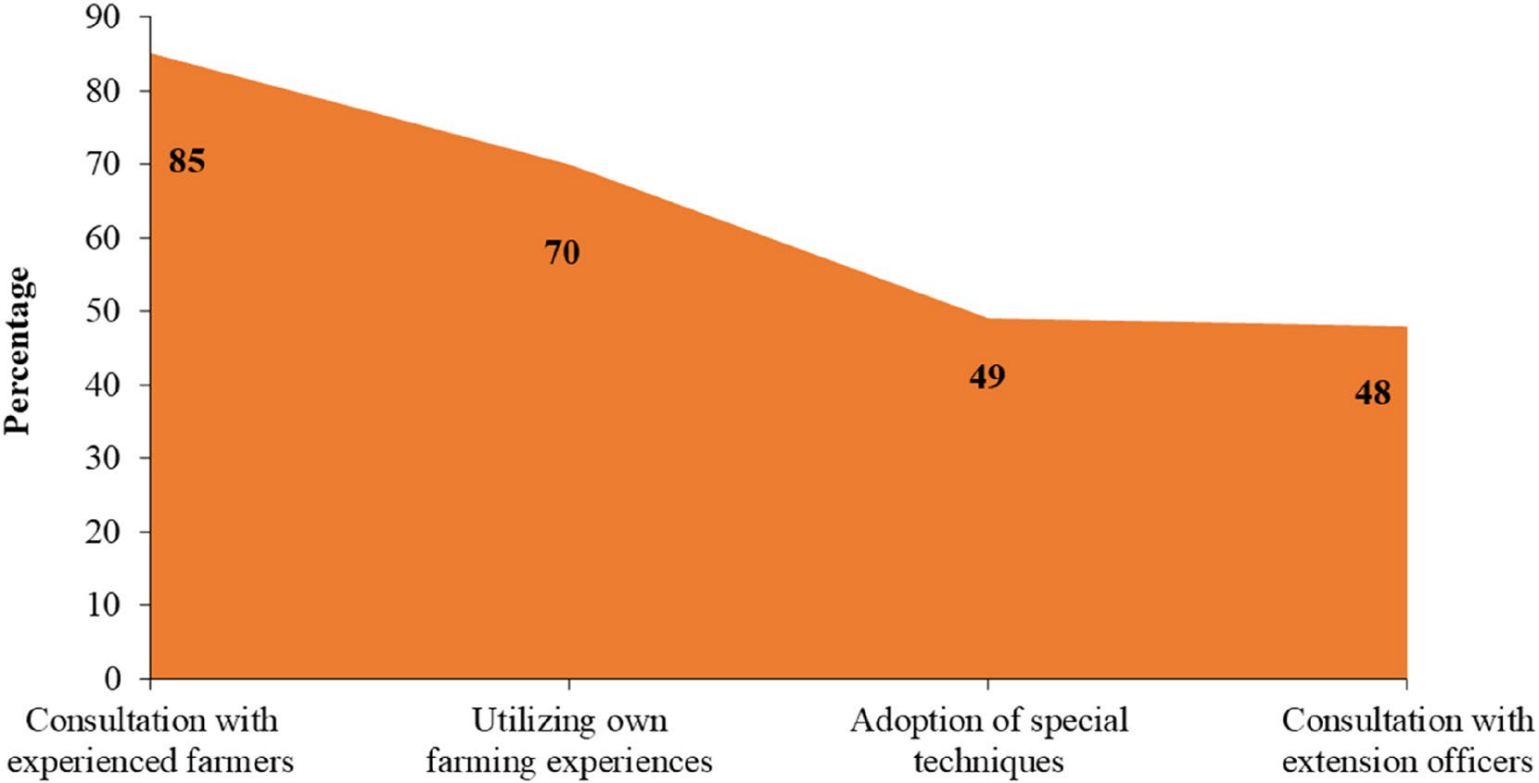
Various ways of managing salinity in cropland.

Several studies have been conducted on salinity management in cropland, and the results of this study are similar to those of (Devkota et al. 2022; Vidal 2019). Devkota et al. (2022) reported that several approaches, such as agronomic, soil, and water management, are needed to reduce salinity, and they strongly suggested that conservation agriculture (CA) could play a role in long‐term agricultural production in salt‐affected irrigated drylands. A study by Singh (2022) revealed that global cases of soil salinity management approaches were conferred by the application of remote sensing and geographic information systems (GISs) in the context of various agrohydroclimatic atmospheres. Therefore, a remote sensing strategy could be a useful tool for assessing plant responses such as evapotranspiration in a variety of salinity settings (Singh 2022). Optimizing the efficacy of irrigation networks, capturing and processing saltwater drainage water, establishing desalination plants, and increasing the level of water that enters aquifers are some of the strategies used to manage the salinity in cropland (Vidal 2019). In addition, growing genetically modified and other salt‐tolerant crops is a more high‐tech solution for managing this problem of salinity (Vidal 2019).

## 4 Conclusion, Recommendations and Future Research Direction

The present study revealed that increasing amounts of inputs and usage of fertilizer increase the volume of rice production and yield. It is also evident from our results that when the level of salinity is low, the yield of rice increases significantly. Furthermore, the use of salt‐tolerant rice varieties and the application of special techniques to manage salinity have a statistically significant effect on technical inefficiency. Furthermore, rice production is lower in areas with high salinity and vice versa. As a result, the profit per decimal in the low‐salinity area is greater than that in the moderate‐ and high‐salinity regions. This study further revealed that farmers tend to migrate from cultivated rice to other crops because salinity causes yield loss, destruction of rice quality, and increased salt concentrations. On the other hand, when a farmer has higher income, more consultation from local extension officers, gains knowledge from other farmers, and receives training, there is a lower likelihood of migrating from cultivated rice to other crops. High labor price, lack of capital, high price of seed, salt land, high price of inputs, lack of extension services, dearth of fertilizer usage, and lack of seed availability are the several major constraints to rice cultivation. There are several techniques followed by farmers in terms of managing salinity in rice land. Among these, the majority of the farmers consulted with other farmers who have more farming experience, followed by utilizing their own farming experience, and consulting with local extension officers.

To maintain the technical efficiency of rice production in areas other than saline areas, farmers need to utilize inputs in an adequate way. Fertilizer needs to be applied to mitigate the effects of salinity. The Fertilizer Recommendation Guide (FRG) of 2024 by the Bangladesh Agricultural Research Council (BARC) could be utilized. In consultation with other farmers and extension officers, frequent field days and farmer consultation days at farmer yards could be organized by the Department of Agricultural Extension (DAE), and various cooperative societies need to be established by the Department of Cooperatives and the Department of Agricultural Marketing (DAM) in Bangladesh. Furthermore, the country's research institutes, namely, the Bangladesh Rice Research Institute (BRRI) and Bangladesh Institute of Nuclear Agriculture (BINA), need to continue developing salt‐tolerant hybrid rice varieties and organized training on management practices for farmers along with extension sectors in Bangladesh. On the other hand, the migration of farmers from rice to other crop cultivation or even professions needs to be checked with priority precaution, as food security highly depends on it for Bangladesh. In doing so, we must focus on reducing salt concentrations in rice‐producing fields and ensuring that no yield or quality loss occurs because of salinity intrusion in coastal areas of Bangladesh. To achieve this goal, we must ensure salt‐tolerant high‐yielding and nutrient‐rich varieties through research and development by BRRI and BINA. Apart from these, frequent and adequate consultation with other experienced farmers and DAE officials in managing salinity could be ensured by the DAE, the Department of Cooperatives, and the DAM in Bangladesh. Furthermore, the execution and coordination of relevant policies require special attention. This study also encourages researchers to study the variety wise rice farming performance of farmers to identify highly salt‐tolerant rice varieties in coastal regions. The impact of farmers' consultation with other farmers and DAEs needs to be estimated and enlightened in future studies.

## Funding

This research was financially supported by Khulna Agricultural University, Khulna‐9100, Bangladesh, and Bangladesh Agricultural Research Institute, Gazipur 1701, Bangladesh.

## Consent

The authors have nothing to report.

## Conflicts of Interest

The authors declare no conflicts of interest.

## Supporting information


**Data S1:** Supporting information.

## Data Availability

All data are available in supplementary raw data file and in all Tables and Figures.
